# Navigation-assisted contralateral sublaminar decompression: a facet-preserving strategy for upper lumbar lesions

**DOI:** 10.3171/2026.4.FOCVID2624

**Published:** 2026-07-01

**Authors:** Hyun Woong Mun, Dong Ah Shin, Seong Yi, Yoon Ha, Keung Nyun Kim, Chang Kyu Lee

**Affiliations:** Department of Neurosurgery, Spine and Spinal Cord Institute, Severance Hospital, Yonsei University College of Medicine, Seoul, Republic of Korea

**Keywords:** unilateral biportal endoscopy, UBE, navigation-assisted surgery, contralateral sublaminar decompression

## Abstract

Navigation-assisted contralateral sublaminar decompression using unilateral biportal endoscopy (UBE) offers a facet-preserving strategy for upper lumbar lesions, particularly in young patients in whom minimizing postoperative instability is critical. The authors present the case of a 34-year-old man with left L2–3 radiculopathy caused by a cystic lesion extending from the subarticular to the foraminal zone. A navigation-assisted contralateral sublaminar UBE approach was selected to achieve adequate neural decompression while preserving facet integrity. Postoperative imaging confirmed complete lesion removal and satisfactory decompression, with minimal contralateral facet loss on quantitative analysis. This video highlights the technical rationale and clinical utility of this approach.

The video can be found here: https://stream.cadmore.media/r10.3171/2026.4.FOCVID2624

## Transcript

This video demonstrates a navigation-assisted contralateral sublaminar decompression as a facet-preserving strategy for upper lumbar lesions.

### 0:24 Clinical Presentation.

The patient is a 34-year-old man who presented with a 2-week history of left leg pain in the L2–3 dermatome. Neurological examination revealed intact motor strength in both lower extremities, with mild sensory tingling in the left L2–3 distribution. Deep tendon reflexes were normal. The patient had no significant past medical history.

### 1:03 Preoperative Imaging.

Lumbar radiographs demonstrated no remarkable findings and no evidence of instability. T2-weighted MRI revealed a 2.4-cm septated cystic lesion with high signal intensity at the L2–3 level, extending from the subarticular to the foraminal zone and compressing the exiting L2 nerve root. On T1-weighted contrast-enhanced images, the lesion demonstrated peripheral rim enhancement.

### 1:38 Differential Diagnosis and Treatment Options.

Based on the imaging characteristics, the differential diagnosis included a soft tissue tumor, such as a discal cyst or synovial facet cyst, as well as a neurogenic tumor. Potential surgical options ranged from conventional microscopic laminectomy and fusion surgery to minimally invasive unilateral biportal endoscopic decompression. For endoscopic decompression, both ipsilateral and contralateral sublaminar approaches were considered in planning the surgical strategy.

### 2:20 Rationale for Surgical Strategy.

The primary goal in this case was to achieve adequate neural decompression while preserving the facet joint, particularly in a young patient.

At the upper lumbar level, the narrow lamina increases the risk of facet joint violation when using an ipsilateral approach.

These anatomical considerations led us to favor a contralateral sublaminar approach, with navigation assistance to ensure accurate localization and safe decompression.

Accordingly, a navigation-assisted contralateral sublaminar approach using unilateral biportal endoscopy was selected.

### 3:06 Patient Positioning.

The patient was positioned prone on a Wilson frame. The O-arm reference frame was firmly fixed to the contralateral thoracic region with double-layer adhesive draping to provide stable navigation reference. The lumbar spine was then prepped and draped in a standard sterile fashion.

### 3:30 Operative Technique.

This is the operative video. Using navigation guidance, the medial margins of the right L2 inferior pedicle and the upper portion of the L3 pedicle are identified. Based on these landmarks, the skin incision sites are marked to ensure an appropriate surgical trajectory. After identification of the spinolaminar junction, ipsilateral laminar drilling is performed. The midline is then confirmed, followed by sublaminar drilling to access the contralateral side. The medial aspect of the contralateral superior articular process was carefully drilled to complete the decompression. Flavectomy was subsequently performed, and the cystic lesion along with ruptured disc fragments was removed. The extent of decompression was confirmed using navigation, followed by meticulous soft tissue handling and hemostasis prior to closure.

### 5:43 Postoperative Clinical Outcome.

Postoperatively, the patient experienced improvement in left leg pain corresponding to the L2–3 dermatome. Histopathological examination revealed degenerated fibrocartilaginous tissue, consistent with a synovial cyst.

### 6:02 Postoperative Imaging.

Postoperative day 2 MRI demonstrated complete removal of the cystic mass with adequate neural decompression.

Quantitative analysis showed minimal loss of the contralateral facet joint, with a reduction rate of 6.6% in facet length and a 9.4% decrease in the cross-sectional area of the facet joint.

### 6:33 Discussion.

Ipsilateral decompression inevitably exposes the medial aspect of the facet joint at the surgical site and may result in substantial facet joint violation. In contrast, contralateral approaches have been shown to be associated with reduced facet joint involvement, particularly when unilateral laminotomy for bilateral lumbar decompression is performed. Previous studies have reported significantly lower rates of facet joint violation with contralateral endoscopic techniques compared to ipsilateral approaches. In this context, navigation assistance facilitates accurate anatomical localization and trajectory planning during contralateral sublaminar decompression, thereby helping to minimize unintended facet joint violation.

### 7:33 Indications, Contraindications, and Limitations.

This approach is best indicated for upper lumbar subarticular–foraminal pathology, where facet preservation is particularly important.

Central disc herniation represents an absolute contraindication. A limitation of this technique is the technical difficulty in directly visualizing the disc space, which may increase the risk of residual pathology and recurrence. In addition, the procedure requires advanced endoscopic proficiency and familiarity with instrument handling, reflecting an associated learning curve.

### 8:18 Navigation Reference Frame Placement.

For navigation-assisted UBE discectomy and ULBD procedures, we typically use a skin-fixed reference frame, as navigation is primarily used for anatomical localization and adjunctive guidance during decompression. In contrast, for endoscopic fusion procedures, a bone-fixed reference frame is preferred to ensure greater stability and accuracy.

In our practice, when performing a left-sided approach, the reference frame is fixed to the right PSIS. For a right-sided approach, due to line-of-sight considerations, the reference frame is fixed to the upper-level spinous process.

**9:02 References^1–10^**

## Acknowledgments

We used ChatGPT (GPT-5.3, OpenAI) for language editing, including improving clarity, grammar, and organization of the text. We reviewed and finalized all content.

## Disclosures

The authors report no conflict of interest concerning the materials or methods used in this study or the findings specified in this publication.

## Author Contributions

Primary surgeon: Lee. Assistant surgeon: Mun. Editing and drafting the video and abstract: Mun. Reviewed submitted version of the work: all authors. Approved the final version of the work on behalf of all authors: Lee. Supervision: Lee.

## Supplemental Information

### Patient Informed Consent

The necessary patient informed consent was obtained in this study.
